# A dynamic model and some strategies on how to prevent and control hepatitis c in mainland China

**DOI:** 10.1186/s12879-019-4311-x

**Published:** 2019-08-16

**Authors:** Wanru Jia, Jie Weng, Cong Fang, Yong Li

**Affiliations:** 1 School of Information and Mathematics, Yangtze University, Jingzhou 434023, China, Nanhuan Road, Jingzhou, 434023 China; 2 College of Mechanical and Vehicle Engineering, Hunan University, Lushan South Road, Changsha, 410082 China; 3 School of Mechanical and Automotive Engineering, South China University of Technology, Wushan Road, Guangzhou, 510641 China; 4 Institute of Applied Mathematics, Yangtze University, Nanhuan Road, Jingzhou, 434023 China

**Keywords:** Hepatitis C, Basic reproduction number, Parameter estimation, Sensitivity analysis, Preventions and control strategies

## Abstract

**Background:**

Hepatitis C virus (HCV) is a leading cause of chronic liver disease. As yet there is no approved vaccine protects against contracting hepatitis C. HCV seriously affects many people’s health in the world.

**Methods:**

In this article, an epidemiological model is proposed and discussed to understand the transmission and prevalence of hepatitis C in mainland China. This research concentrates on hepatitis C data from Chinese Center for Disease Control and Prevention (China’s CDC). The optimal parameters of the model are obtained by calculating the minimum chi-square value. Sensitivity analyses of the basic reproduction number and the endemic equilibrium are conducted to evaluate the effectiveness of control measures.

**Results:**

Vertical infection is not the most important factor that causes hepatitis C epidemic, but contact transmission is. The proportion of acute patients who are transformed into chronic patients is about 82.62%. The possibility of the hospitalized patients who are restored to health is about 76.24%. There are about 92.32% of acute infected are not treated. The reproduction number of hepatitis C in mainland China is estimated as approximately 1.6592.

**Conclusion:**

We find that small changes of transmission infection rate of acutely infected population, transmission infection rate of exposed population, transition rate for the acutely infected, and rate of progression to acute stage from the exposed can achieve the purpose of controlling HCV through sensitivity analysis. Finally, based on the results of sensitivity analysis, we find out several preventions and control strategies to control the Hepatitis C.

## Background

Hepatitis C virus(HCV) seriously affects lots of people’s health in the world. Recently (18 July, 2018), the World Health Organization (WHO) estimates that approximately 71 million people have chronic hepatitis C virus (HCV) infection worldwide and approximately 399,000 people die each year after HCV diagnosis, mostly from cirrhosis and hepatocellular carcinoma (HCC) [1]. An estimated 3.5 million people in the United States (US) has chronic hepatitis C [2]. In 2016, there are 18,153 hepatitis C-related deaths in the US which is lower than from 2012 to 2015 (18,650 to 19,629) [3]. In the European region, approximately 14 million people are chronically infected with HCV, representing about 20% of the global burden of disease due to HCV infection [4]. The areas where have the highest reported prevalence rates locate in Africa and Asia, and China in the Asia whose citizens account for about one fifth of the world’s populace, has a reported seroprevalence about 3% [5–7].

HCV was discovered in 1989 by Choo et al. [8, 9], it is a small, enveloped, single-stranded ribonucleic acid (RNA) virus, which be part of the Flaviviridae family. Hepatitis C is an infectious disease caused by HCV which basically affect the liver. The spread ways of the virus are blood transmission, sexual transmission and mother-to-child transmission, but the leading way is blood transmission, such as sharing injection equipment, inputing the contaminated blood or blood products, tattooing [10]. As yet there is no approved vaccine to protect against contracting hepatitis C. The focus of prevention efforts should be safe blood supply in the developing world, safe injection practices in health care and other settings, and less amount of people who inject drugs [11]. In those persons who do develop symptoms, the mean time period from exposure to symptom onset is 3–12 weeks (range: 2–24 weeks) [12, 13]. HCV infection has both acute and chronic forms, the incubation for chronic HCV can be between 14 to 180 days [12]. Acute hepatitis C infection is hard to diagnose, because 70% to 80% of the patients are symptomless [13, 14]. Most of them are unconscious of their exposure to HCV, and fail to get diagnosed in time until the occurring of the secondary symptoms to the liver. Some studies show, however, the acute infection phase is very impressionable to treatment, so it is an unique occasion to prevent the evolution of chronic infection [15]. Chronic hepatitis C can bring about cirrhosis and HCC. The average rate of progression of the disease is extremely slow. Using data collected in Japan, investigators estimate that, following acute infection, chronic hepatitis could be ensured 13.7±10.9 years later, chronic active hepatitis could be ensured 18.4±11.2 years later, cirrhosis of the liver could be ensured 20.6±10.1 years later, and hepatocellular carcinoma could be ensured in 28.3±11.5 years [16, 17].

Some mathematical models were used to analyze the spread of hepatitis C disease and come up with some effective strategies. Martcheva M and Castillo-Chavez C [10] considered an epidemiological model with a chronic infectious phase and variable population size, and the analysis consequences revealed that treatment strategies directed forward speeding up the transition from acute to chronic stage in effect conduce to the eradication of the diseases. This model was extended by Das P et al. [15] who incorporate the immune class and was also extended by Yuan J [18] who consider the latent period. Imran M [19] formulated epidemic models of hepatitis C considering an isolation class and analyzed the effects of the isolation class on the transmission dynamics of the disease. Mathematical modeling of hepatitis C treatment for injecting drug users (IDUs) were studied in [20–22] where the treated individuals are supposed not to infect the susceptible individuals. Lately, there are some researches [23, 24] about hepatitis C epidemic cases which suggest some measures to control hepatitis C infection continental China. But these models did not consider the vertical infection. It is not effortless to diagnosis due to the shortage of the residents’ consciousness and the characteristics of the patients with hepatitis C, so it is probable that patients will transmit HCV to their children.

The aim of this work is to use mathematical modelling to investigate the influences of hepatitis C, then probe and draw some conclusions about effective policy. The organization of this paper is as follows. In the next section, an epidemic model for hepatitis C is proposed to prevent and control the infectious disease. Then we acquire its optimal parameter values by Matlab tool fmincon and compare the reported data and simulative results. Sensitivity analyses of the basic reproduction number and the endemic equilibrium are performed in “Results” section. After that, discussion on the model parameters and the main factors affecting the spread of hepatitis C in “Discussion” section, and we end this article with how to control the hepatitis C in “Conclusions” section.

## Methods

### Data

We have found clinical cases of hepatitis C in China every month from 2011 to 2016 from the China Center for Disease Control and Prevention (China’s CDC), which is a public welfare institution organized by the Chinese government to implement state-level disease prevention and control and public health technology management and services. China’s CDC conducts monthly statistics on patients infected with hepatitis C virus in mainland China (i.e., except Hong Kong, Macao and Taiwan) [25] including gender, occupation, date of birth, address, date of onset, date of diagnosis, especially the classification of the disease, which is marked as a clinically diagnosed case.

In general, it is unreasonable to determine HCV infections just by relying on HCV antibody positive which just means you were infected before. To determine whether infected with HCV, HCV-RNA test needs to be done. Once the HCV-RNA test results indicate that the outpatient is infected with the hepatitis C virus, he or she will need hospitalization. In the case of ignoring the patient’s home treatment, we believe that the data provided by the China’s CDC is the number of hospitalizations.

By producing re-sampling a larger artificial data set, which is generated based on the existing limited reported monthly data, using the linspace function from Matlab (the Mathworks, Inc.), we interpolate the 12-month data and turn into 365-day data. In order to keep the total number of data, the interpolation formula of each year as following:

$$\begin{array}{@{}rcl@{}} {\hat D_{2}}({t_{j}}) = \frac{{{D_{2}}({t_{j}})\sum\limits_{i = 1}^{12} {{D_{1}}({s_{i}})} }}{{\sum\limits_{j = 1}^{365} {{D_{2}}({t_{j}})} }},j = 1,2, \cdots,365,  \end{array} $$

where, *D*_1_(*s*_*i*_),*i*=1,2⋯,12, denote the 12-month actual data, *D*_2_(*t*_*j*_),*j*=1,2⋯,365, denote the 365-day data after the interpolation. ${\hat D_{2}}({t_{j}}), j=1,2 \cdots, 365,$ denote the 365-day data after the zoom. With the aid of linear interpolation, we will obtain more useful data, and the fit results will be better. We still give a comparison chart for each month’s case data and simulative data.

### Model formulation

In order to study the epidemic of hepatitis C in China, we consider the hepatitis C model is homogeneous mixing-an individual has an equal chance of contacting any individual among the population, by ignoring the impacts of the space structure and seasonal changes to simulate the data year after year, and we assume that natural birth rate is equal to natural mortality.

The mathematical model for hepatitis C to understand the transmission dynamics and prevalence consists of a system of ordinary differential equations, where population is divided into six subgroups: susceptible *S*(*t*), exposed *E*(*t*) (defined as not infected but infectious), acute infection *I*_*a*_(*t*), chronic infection *I*_*c*_(*t*), treated *T*(*t*) and recovered *R*(*t*) individuals. The total population size is denoted by *N*(*t*)=*S*(*t*)+*E*(*t*)+*I*_*a*_(*t*)+*I*_*c*_(*t*)+*T*(*t*)+*R*(*t*).

**Fig. 1:**
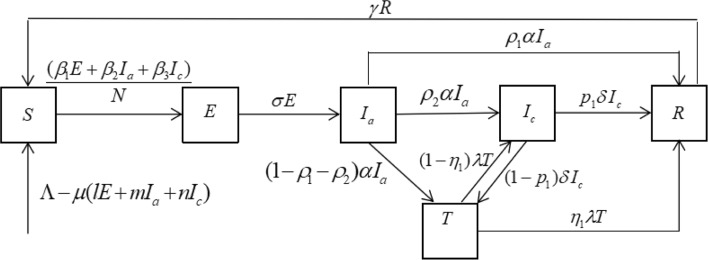
Flow chart of compartments of hepatitis C model

New susceptible individuals enter into the *S* compartment with a recruitment rate *Λ*. Let *μ* be the natural birth and death rate of the population. By the influence of their parents, generations of the individuals in the *E*(*t*),*I*_*a*_(*t*),*I*_*c*_(*t*) may be infected with HCV at rate of *l,m*,*n*, respectively. This is what is called vertical infection. Susceptible individuals are infected by contacting with patients in the *E*(*t*),*I*_*a*_(*t*),*I*_*c*_(*t*) compartments at rates of *β*_1_,*β*_2_,*β*_3_, respectively. Once infected, the individuals move into the exposed compartment (*E*) and then progress to the acute stage at a rate of *σ*. In the acute stage, the individuals may die at rate of *d*_1_. Let *α* be the transition rate for the acutely infected individuals. In the conversion of acute infection, the individuals will restore health relying on their own immune system with the ratio *ρ*_1_, progress to the chronic stage with the ratio *ρ*_2_, go to the hospital for treatment with the ratio 1−*ρ*_1_−*ρ*_2_. At the same, the individuals may die at rate of *d*_2_ in the chronic stage. Let *δ* be the transition rate for the chronically infected individuals. In the conversion of chronic infection, the individuals will restore health relying on their own immune system with the ratio *p*_1_, go to the hospital for treatment with the ratio 1−*p*_1_. Individuals in the treated compartment (*T*) who have the transition rate of *λ*, succeed in clearing HCV and move to the recovered compartment (*R*) with the ratio *η*_1_, while the others fail and move back to the chronic stage with the ratio 1−*η*_1_. Individuals in the *R* compartment lose their immunity and eventually return to the susceptible compartment (*S*) at rate of *γ*. The schematic flow diagram illustrating the transmission dynamics of the HCV infection with treatment are illustrated in Fig. 1. And the biological meanings and acceptable ranges of all parameters are listed in Table 1.

**Table 1:** Model parameters and their interpretations

Parameter	Description
*μ*	Natural birth or death rate
*Λ* in (10^2^,10^6^)	Recruitment rate
*l* in (0, 0.1)	Transmission rate of the exposed generation
*m* in (0, 0.1)	Transmission rate of the acute infection generation
*n* in (0, 0.1)	Transmission rate of chronic infection generation
*β*_1_ in (0, 0.1)	Transmission infection rate of exposed population
*β*_2_ in (0, 0.1)	Transmission infection rate of acutely infected population
*β*_3_ in (0, 0.1)	Transmission infection rate of chronically infected population
*γ* in (0, 1/30)	Remove rate from recovered to susceptible
*σ* in (0, 0.05)	Rate of progression to acute stage from the exposed
*α* in (0, 0.5)	Transition rate for the acutely infected
*d*_1_ in (0, 0.01)	The mortality of acutely infected population
*d*_2_ in (0, 0.01)	The mortality of chronically infected population
*ρ*_1_ in (0, 0.1)	The proportion of natural recovered from acutely infected population
*ρ*_2_ in (0.5, 1)	The proportion of chronic infection from acutely infected population
*δ* in (0, 0.1)	Transition rate for the chronically infected
*η*_1_ in (0, 1)	The proportion of recovered from treated population
*λ* in (0, 1)	Transition rate for the treated
*p*_1_ in (0, 1)	The proportion of natural recovered from chronically infected population
${\mathcal {R}_{0}}$	The number of infected during the initial patient’s infectious (not sick) period

The model is represented by the following system of ordinary differential equations:

1 $$\begin{array}{@{}rcl@{}} {\begin{aligned} \left\{ \begin{array}{l} \frac{{dS}}{{dt}} \,=\, \Lambda \,-\, \mu lE \,-\, \mu m{I_{a}} \,-\, \mu n{I_{c}} \,-\, \frac{{\left({{\beta_{1}}E + {\beta_{2}}{I_{a}} + {\beta_{3}}{I_{c}}} \right)S}}{N} \,+\, \gamma R \,-\, \mu S,\\ \frac{{dE}}{{dt}} \,=\, \frac{{\left({{\beta_{1}}E + {\beta_{2}}{I_{a}} + {\beta_{3}}{I_{c}}} \right)S}}{N} \,-\, \sigma E - \mu E + \mu lE + \mu m{I_{a}} + \mu n{I_{c}},\\ \frac{{d{I_{a}}}}{{dt}} \,=\, \sigma E - \alpha {I_{a}} - \mu {I_{a}} - {d_{1}}{I_{a}},\\ \frac{{d{I_{c}}}}{{dt}} = {\rho_{2}}\alpha {I_{a}} - \delta {I_{c}} + \left({1 - {\eta_{1}}} \right)\lambda T - \mu {I_{c}} - {d_{2}}{I_{c}},\\ \frac{{dT}}{{dt}} = \left({1 - {\rho_{1}} - {\rho_{2}}} \right)\alpha {I_{a}} + \left({1 - {p_{1}}} \right)\delta {I_{c}} - \lambda T - \mu T,\\ \frac{{dR}}{{dt}} = {p_{1}}\delta {I_{c}} + {\rho_{1}}\alpha {I_{a}} + {\eta_{1}}\lambda T - \gamma R - \mu R. \end{array} \right.  \end{aligned}} \end{array} $$

The biologically feasible region $\Omega = \{ (S,E,{{I}_{a}},{{I}_{c}},T,R) \in \mathbb {R}_ +^{6}:S + E + {I}_{a} + {I}_{c} + T + R < \frac {\Lambda }{\mu }\} $ is a positively invariant set of system (1).

The basic reproduction number (${\mathcal {R}_{0}}$) represents the number of infected during the initial patient’s infectious (not sick) period. What this threshold will do determine whether a disease will die out (if ${\mathcal {R}_{0}}< 1$) or become epidemic (if ${\mathcal {R}_{0}}> 1$). For models with complex dynamics, ${\mathcal {R}_{0}}< 1$ is not the only condition to guarantee that the disease is extinct, but the smaller the better. Following Van den Driessche P and Watmough J [26], the basic reproduction number for the model (1) is given by the formula:

$$\begin{array}{@{}rcl@{}} {\begin{aligned} \begin{array}{l} {\mathcal{R}_{0}} = \left({{\beta_{1}} + \mu l} \right)\frac{1}{{\sigma + \mu }} + \left({{\beta_{2}} + \mu m} \right)\frac{\sigma }{{\left({\sigma + \mu} \right)\left({\alpha + \mu + {d_{1}}} \right)}} + \left({{\beta_{3}} + \mu n} \right)A\\ ={\mathcal{R}_{01}}+{\mathcal{R}_{02}}+{\mathcal{R}_{03}}+{\mathcal{R}_{04}}+{\mathcal{R}_{05}}+{\mathcal{R}_{06}}, \end{array} \end{aligned}} \end{array} $$

where, ${\mathcal {R}_{01}}=\frac {{{\beta _{1}}}}{{\sigma + \mu }}, {\mathcal {R}_{02}}=\frac {{{\beta _{2}}\sigma }}{{\left ({\sigma + \mu } \right)\left ({\alpha + \mu + {d_{1}}} \right)}}, {\mathcal {R}_{03}}={\beta _{3}}A, {\mathcal {R}_{04}}=\frac {{\mu l}}{{\sigma + \mu }}, {\mathcal {R}_{05}}=\frac {{\mu m\sigma }}{{\left ({\sigma + \mu } \right)\left ({\alpha + \mu + {d_{1}}} \right)}}, {\mathcal {R}_{06}}=\mu nA, A = \frac {{\alpha \sigma \left [ {{\rho _{2}}\left ({\lambda + \mu } \right) + \lambda \left ({1 - {\eta _{1}}} \right)\left ({1 - {\rho _{1}} - {\rho _{2}}} \right)} \right ]}}{{\left ({\sigma + \mu } \right)\left ({\alpha + \mu + {d_{1}}} \right)\left [ {\left ({\delta + \mu + {d_{2}}} \right)\left ({\lambda + \mu } \right) - \delta \lambda \left ({1 - {p_{1}}} \right)\left ({1 - {\eta _{1}}} \right)} \right ]}}.$

${\mathcal {R}_{01}}, {\mathcal {R}_{02}}$ and ${\mathcal {R}_{03}}$ represent the average numbers of the infected individuals by a single exposed, acute infection or chronic infection individual in a fully susceptible population, respectively. ${\mathcal {R}_{04}}, {\mathcal {R}_{05}}$ and ${\mathcal {R}_{06}}$ represent the average numbers of the infected infants by the exposed, acute infection or chronic infection parents, respectively. They represent the contributions of the 6 HCV transmission ways to the the basic reproduction number ${\mathcal {R}_{0}}$.

### Parameter estimation

In this section, we first use model (1) to simulate the reported hepatitis C data of China from January 2011 to December 2016 to predict the trend of the disease and seek of some preventions and control measures. The data are obtained mainly from epidemiologic bulletins published by the China’s CDC [25]. Assume that the person’s natural death follows a uniform distribution, then natural death rate is calculated as *μ*=1/(74.83×365)=3.6613×10^−5^, since life expectancy is 74.83 years old between 2011 to 2016 in China [27]. From Shen M [24], the range of the transmission rates ${\tilde \beta _{\mathrm {i}}}, i=1,2,3$ is [2.0846,3.0769]×10^−11^, and those annual transmission rates are bilinear. Total population is about 1.35×10^9^ in China between 2011 to 2016 [27], We chose 80% of the population as the sampled population, and denote as $\tilde N=1.08\times {10}^{9}$. So we estimate the standard rate ${\hat \beta _{i}} = {\tilde \beta _{i}}\tilde N \in [0.0225,0.0323], i=1,2,3$. The values of *β*_1_,*β*_2_ and *β*_3_ in model (1) are chosen randomly in this interval.

Then, we have to estimate the other 15 parameters and 6 initial values every year through calculating the minimum sum of chi-square [28, 29]

$$\begin{array}{@{}rcl@{}} J(\theta) = \sum\limits_{i = 1}^{72} {\frac{{{{(T({t_{i}}) - \hat T({t_{i}}))}^{2}}}}{{\hat T({t_{i}})}}}  \end{array} $$

with the MATLAB (the Mathworks, Inc.) tool fmincon that is a part of optimization toolbox. Where, *T*(*t*_*i*_),*i*=1,2,⋯,72 show the true value each month, ${\hat T({t_{i}})}, i=1,2,\cdots,72$ show the estimated value each month. Fmincon function is a Matlab function for solving the minimum value of constrained nonlinear multivariate function. Fmincon implements four different algorithms: interior point, sequence quadratic program (SQP), active set, and trust region reflective. In this paper, we choose the SQP algorithm to solve the optimal solution of model (1). MATLAB SQP method is divided into three steps: firstly, update the Lagrangian Hessian matrix, then solve the quadratic programming problem, and finally calculate the one-dimensional search and objective function.

According to the epidemiological characteristics of hepatitis C and the biological significance of the parameters, we set the lower and upper boundaries of each parameter, as shown in Table 1. Although the outbreak of hepatitis C is not seasonal, it still has a certain periodicity. Our model does not have a periodic solution, so we can only simulate the annual parameter values separately. The simulated annual parameter values are shown in Table 2. Taking year as the research unit, the parameters of the model (1) vary from year to year because of the annually different natural conditions and environmental factors, but the same parameters are not significantly different in different years.

**Table 2:** Annual simulation values of the parameters between 2011 and 2016 and ${\mathcal {R}_{0}}$

parameter	2011	2012	2013	2014	2015	2016	Source
*Λ*	8.15×10^2^	1.58×10^4^	100	113	2.05×10^4^	7.03×10^3^	Estimation
*l*	1×10^−10^	4.41×10^−5^	1×10^−10^	0.1	0.1	0.1	Estimation
*m*	1×10^−10^	1.03×10^−3^	1×10^−10^	0.1	1.06×10^−3^	0.1	Estimation
*n*	4.89×10^−6^	2.78×10^−5^	1×10^−10^	0.1	9.46×10^−2^	0.1	Estimation
*β*_1_	2.23×10^−2^	2.24×10^−2^	2.23×10^−2^	2.23×10^−2^	2.23×10^−2^	2.23×10^−2^	[24]
*β*_2_	2.46×10^−2^	3.10×10^−2^	2.46×10^−2^	2.46×10^−2^	2.46×10^−2^	2.46×10^−2^	[24]
*β*_3_	2.20×10^−2^	2.34×10^−2^	2.20×10^−2^	2.20×10^−2^	2.20×10^−2^	2.20×10^−2^	[24]
*γ*	8.45×10^−4^	1.01×10^−3^	10^−6^	8.90×10^−4^	3.81×10^−5^	2.11×10^−3^	Estimation
*σ*	2.88×10^−2^	5.00×10^−2^	3.63×10^−2^	3.03×10^−2^	3.33×10^−2^	2.72×10^−2^	Estimation
*α*	1.85×10^−2^	5.30×10^−2^	2.20×10^−2^	1.40×10^−2^	1.45×10^−2^	1.62×10^−2^	Estimation
*d*_1_	10^−2^	5.08×10^−3^	10^−2^	10^−2^	9.89×10^−3^	10^−2^	Estimation
*d*_2_	10^−2^	9.53×10^−3^	10^−2^	10^−6^	10^−2^	2.50×10^−3^	Estimation
*ρ*_1_	0.1	8.20×10^−2^	0.1	0.1	0.1	0.1	Estimation
*ρ*_2_	0.82	0.80	0.84	0.83	0.80	0.87	Estimation
*δ*	0.1	8.30×10^−2^	0.1	9.46×10^−2^	0.1	0.1	Estimation
*η*_1_	0.46	0.34	0.90	0.95	0.97	0.96	Estimation
*λ*	0.99	0.94	0.96	0.96	0.91	0.92	Estimation
*p*_1_	0.93	0.82	0.94	0.91	0.90	0.93	Estimation
${\mathcal {R}_{0}}$	1.75	1.21	1.50	1.87	1.77	1.86	Calculated

**Fig. 2:**
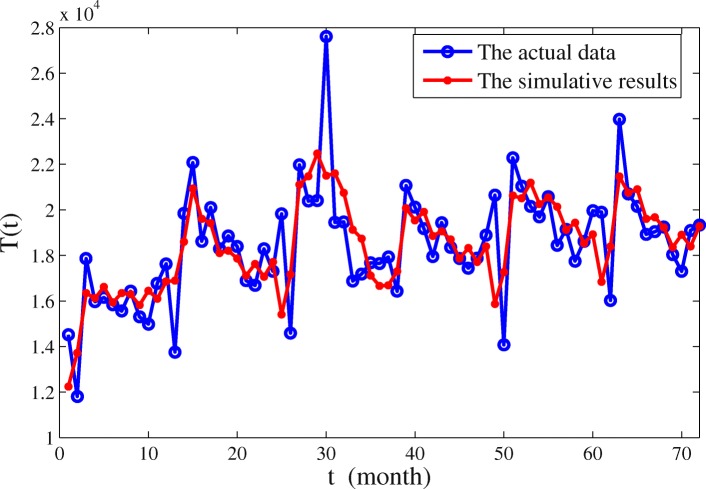
The comparison between the reported hepatitis C in China from 2011 to 2016 and the simulation of model (1)

The values of the various parameters in Table 2 are in days. We calculated the numbers of the treated in each month of each year according to the optimal simulation parameters, then, compared it with the reported hepatitis C data in China from 2011 to 2016 per month. We use two broken line diagrams, as shown in Fig. 2. The data presented in Fig. 2 refers to the clinical data from China’s CDC, denoted by *T*. And the numerical results are found to be a good match with the data of hepatitis C in China from 2011 to 2016 except one point which represent the number of treated patients in June 2013. So we guess the abnormality of this data could be related to the emergence of new avian influenza H7N9 [30] and the 7.8-magnitude earthquake in Ya’an, Sichuan province [31] in China in April of that year. Our model is based on the ideal state, without considering the impact of unexpected events, so the model is not able to capture that outbreak.

We found the optimal parameter values and the initial values of the model in 2011 after continuous debugging, then, using the optimal parameter values of the model in 2011 as the starting value, we have found the optimal parameter values of each subsequent year through continuous simulation. Where, the optimal values of parameters are listed in Table 2, and the each initial condition from 2011 to 2016 is fixed as (4.23×10^7^,3.81×10^5^,10^2^,7.46×10^4^,4.69×10^2^,4.08×10^7^),(3.66×10^7^,8.54×10^4^,1.16×10^2^,3.08×10^4^,4.36×10^2^,1.10×10^7^),(9.01×10^7^,4.18×10^5^,1.18×10^2^,10^5^,6.53×10^2^,4.80×10^7^),(7.07×10^7^,4.64×10^5^,6.51×10^4^,6.12×10^4^,5.77×10^2^,8.98×10^7^),(5.36×10^7^,3.48×10^5^,9.10×10^4^,6.17×10^4^,6.98×10^2^,4.52×10^7^),(2.66×10^7^,9.71×10^5^,10^2^,8.81×10^4^,6.46×10^2^,5.83×10^7^). Here, we also calculate the basic reproduction number each year. The average value of the 6 basic reproduction numbers is estimated as approximately 1.6592. Using the optimal parameters of 2015, one also calculates ${\mathcal {R}_{01}}=0.6701, {\mathcal {R}_{02}}=1.0050, {\mathcal {R}_{03}}=0.0958, {\mathcal {R}_{04}}=1.0992\times {10}^{-4}, {\mathcal {R}_{05}}=1.5888\times {10}^{-6}, {\mathcal {R}_{06}}=1.5094\times {10}^{-5}$. Hence, vertical infection is not the main factor that cause hepatitis C epidemic, but transmission of HCV from exposed and infection to others is the most important factor. We will discuss this argument again in next sections. However, because of China’s big population base, vertical infection is still worthy of our attention.

## Results

### Sensitivity analysis of ${\mathcal {R}_{0}}$

In this section we performed a sensitivity analysis of the basic reproduction number to determine several parameters that have the most influential parameters on the prevalence and transmission of hepatitis C. Sensitivity analysis is a useful tool to identify how closely input parameters are related to predictor parameters and it helps to determine level of change necessary for an input parameter to find the desire value of a predictor parameter [32, 33]. If a small change in a parameter can cause a large change in the number of the basic reproduction number, then this parameter is called a sensitivity factor, otherwise called an insensitive factor.

In this section, following Samsuzzoha M’s [32] method, we used the 2015 simulated parameter values to perform a sensitivity analysis of the basic reproduction number, thus we can put some effective control strategies of HCV. The sensitivity indices of each parameter to the basic reproduction number ${\mathcal {R}_{0}}$ are shown in Table 3.

**Table 3:** Sensitivity indices of ${\mathcal {R}_{0}}$, the corresponding % changes needed to affect a 1% decrease/increase in the value of ${\mathcal {R}_{0}}$

Parameter	Sensitivity indices of ${\mathcal {R}_{0}}$	Corresponding % changes
*β*_2_	+0.5573	−1.7945
*β*_1_	+0.3879	−2.5777
*σ*	−0.3575	+2.7973
*α*	−0.2938	+3.4031
*β*_3_	+0.0526	−19.0167
*ρ*_2_	+0.0510	−19.6182
*δ*	−0.0475	+21.0344
*η*_1_	−9.5265×10^−3^	+104.9707
*l*	+ 1.9356×10^−3^	−516.6356
*p*_1_	−1.3947×10^−3^	+717.0208
*n*	+ 2.5209×10^−4^	−3.9669×10^3^
*ρ*_1_	−2.0715×10^−4^	+ 4.8274×10^3^
*m*	+ 2.6796×10^−5^	−3.7319×10^4^
*λ*	+ 3.8024×10^−7^	−2.6299×10^6^

We can observe that *β*_2_,*β*_1_,*β*_3_,*ρ*_2_, *l*, *n*, *m*, *λ*, (*σ*,*α*,*δ*,*η*_1_,*p*_1_,*ρ*_1_) have positive (negative) impacts on ${\mathcal {R}_{0}}$. The sensitivity indices and corresponding % value needed to affect a 1% decrease in ${\mathcal {R}_{0}}$ are shown in Table 3 (e.g., in order to decrease the value of ${\mathcal {R}_{0}}$ by 1% it is necessary to decrease the value of *β*_2_ by 1.7945% or increase the value of *σ* by 2.7973%.) The greater absolute value of the sensitivity index, the more sensitive the parameter is to ${\mathcal {R}_{0}}$. Therefore, the most sensitive parameter for ${\mathcal {R}_{0}}$ is *β*_2_ followed by *β*_1_,*σ*,*α*,*β*_3_,*ρ*_2_,*δ*,*η*_1_, *l*, *p*_1_, *n*, *ρ*_1_, *m*, *λ*. From Table 3, we can see that parameters *l*, *m*, *n* can be negligible on the influence of the basic reproduction number (${\mathcal {R}_{0}}$) compared with the most sensitive parameters *β*_2_,*β*_1_,*σ*,*α*. Hence, vertical infection is not the main factor that cause hepatitis C epidemic in China. In the “Conclusions” section, we will put forward some specific human intervention measures according to the results.

### Sensitivity analysis of the endemic equilibrium

In this section, we do a sensitivity analysis of the endemic equilibrium to determine the relative importance of the different parameters which are responsible for the prevalence of equilibrium disease. Using the method from Samsuzzoha M [32], we calculate the sensitivity indices of the endemic equilibrium. The relevant detail calculation is shown in Appendix, and the parameter values are shown in Table 4 by using the parameters values of 2015 given in Table 2. We can see that: the most sensitive parameter for *S*^∗^ is *α* followed by *p*_1_,*β*_2_,*ρ*_2_,*β*_1_,*σ*,*δ*,*η*_1_,*ρ*_1_,*β*_3_,*l,n*,*m* and *λ*. The most sensitive parameter for *E*^∗^ is *σ* followed by *β*_2_,*β*_1_,*α*,*p*_1_,*δ*,*β*_3_,*ρ*_1_,*ρ*_2_,*η*_1_,*l,n*,*λ* and *m*. The most sensitive parameter for ${I}_{a}^{*}$ is *α* followed by *β*_2_,*β*_1_,*σ*,*p*_1_,*δ*,*β*_3_,*ρ*_1_,*ρ*_2_,*η*_1_,*l,n*,*λ* and *m*. The most sensitive parameter for ${I}_{c}^{*}$ is *ρ*_2_ followed by *δ*,*β*_2_,*β*_1_,*σ*,*η*_1_,*α*,*p*_1_,*β*_3_,*ρ*_1_,*l,n*,*λ* and *m*. The most sensitive parameter for *T*^∗^ is *β*_2_ followed by *β*_1_,*σ*,*ρ*_1_,*λ*,*ρ*_2_,*η*_1_,*α*,*p*_1_,*β*_3_,*δ*,*l,n* and *m*. The most sensitive parameter for *R*^∗^ is *β*_2_ followed by *p*_1_,*ρ*_2_,*β*_1_,*σ*,*α*,*η*_1_,*ρ*_1_,*β*_3_,*δ*,*l,n*,*λ* and *m*. For the above analysis, we can see that the sensitivity of the four parameters *β*_1_,*β*_2_,*α*,*σ* are at the top of the sensitivity indices of the endemic equilibrium, especially for ${I}_{a}^{*}$, and the sensitivity of *ρ*_2_,*δ*,*β*_2_,*β*_1_,*σ* are at the top of ${I}_{c}^{*}$. So if we want to reduce the number of cases, we can propose specific preventive control measures from these parameters in the “Conclusions” section.

**Table 4:** Sensitivity indices of the endemic equilibrium

parameter	*S* ^∗^	*E* ^∗^	${I}_{a}^{*}$	${I}_{c}^{*}$	*T* ^∗^	*R* ^∗^
*l*	+1.20×10^−4^	−7.96×10^−5^	−7.96×10^−5^	−8.15×10^−5^	−4.07×10^−4^	−1.33×10^−4^
*m*	+1.74×10^−6^	−1.15×10^−6^	−1.15×10^−6^	−1.18×10^−6^	−5.89×10^−6^	−1.92×10^−6^
*n*	+1.65×10^−5^	−1.09×10^−5^	−1.09×10^−5^	−1.12×10^−5^	−5.59×10^−5^	−1.82×10^−5^
*β*_1_	+0.4145	−0.2740	−0.2740	−0.2806	−1.4024	−0.4573
*β*_2_	+0.6216	−0.4109	−0.4109	−0.4209	−2.1034	−0.6859
*β*_3_	+0.0592	−0.0392	−0.0392	−0.0401	−0.2004	−0.0654
*σ*	−0.4131	+1.2710	+0.2710	+0.2776	+1.3872	+0.4524
*α*	−0.9452	+0.2684	+0.8617	−0.1417	−0.7081	−0.2309
*ρ*_1_	+0.0737	−0.0049	−0.0049	+0.0020	+1.1541	+0.0753
*ρ*_2_	−0.4462	−0.0035	−0.0035	−0.9918	+0.9775	−0.5742
*δ*	−0.1720	+0.0436	+0.0436	+0.9510	−0.1872	−0.0609
*η*_1_	+0.0838	+6.51×10^−4^	+6.51×10^−4^	+0.1863	+0.8164	+0.1078
*λ*	+3.86×10^−7^	−3.77×10^−6^	−3.77×10^−6^	−4.10×10^−6^	+0.9999	−1.30×10^−5^
*p*_1_	−0.6518	+0.0453	+0.0453	+0.0464	+0.2317	−0.6588

## Discussion

From Table 2, according to discuss the arithmetic means of parameters of our model, we have some conclusions as follows: $\bar l = 5.00\%$ (e.g., $\bar l = \frac {1}{6}\sum \limits _{i = 2011}^{2016} {{l_{i}}}$, the method of calculating the average value of other parameters is the same.), $\bar m = 3.37\%, \bar n = 4.91\%$, these suggest that the probabilities of exposed, the acute and the chronic patients spread virus to their kids on hepatitis C are about 5.00%, 3.37% and 4.91%, respectively. $\bar {\rho _{1}} = 9.70\%$, it shows that the proportion of patients who recover naturally in all acute patients is about 9.70%. $\bar {\rho _{2}} = 82.62\%$, it shows that the proportion of acute patients who turned into chronic patients is about 82.62%. From Chen SL [13], approximately 75*%*−85*%* of infected patients do not clear the virus in 6 months, and become chronic hepatitis patients. $1-\bar {\rho _{1}} -\bar {\rho _{2}} = 7.68\%$, it indicates that the proportion of acute patients who are treated in hospital is about 7.68%. This result is similar to that of Cox AL’s [34], he denotes that 95% of infected are not treated. $\bar {\eta _{1}} = 76.24\%$, it suggests that the proportion of the resident patients who can recover is about 76.24%. From Seeff LB [35], about 80% of HCV-infected individuals seem to be no progression to end-stage liver disease, but 20% who get histologic fibrosis and cirrhosis will develop into serious end-stage liver disease. And in our paper, $1-\bar {\eta _{1}} = 23.76\%$, it suggests that the proportion of the resident patients who failed to recover is about 23.76%, while we don’t consider that chronic patients develop histologic fibrosis and cirrhosis, which will be our follow-up work. $1/{{\bar \gamma }}\approx 1226.03$ days, i.e., 3.36 years, it suggests that the average time that the antibody disappear is about 3.36 years. $1/{{\bar \sigma }}\approx 29.12$ days, it shows that the average incubation time is about 29.12 days. $1/{{\bar \delta }}\approx 10.39$ days, it shows that the average period of chronic patients deciding whether to be treated or not is about 10.39 days. Then, these conclusions have been conformed to the actual situation [1, 25].

According to the values of the parameters and sensitivity analysis of the basic reproduction number and the endemic equilibrium, we can find that vertical infection is not the primary cause of hepatitis C epidemic in China, the reasons are as follows:

(1)${\bar R_{04}} = 6.07 \times {10^{- 5}}, {\bar R_{05}} = 4.9 \times {10^{- 5}}, {\bar R_{06}} = 8.85 \times {10^{- 6}}$, these represent the average contribution from the generation of the exposed, the acute and the chronic to the basic reproduction number (${\mathcal {R}_{0}}$), respectively. We can observe that vertical infection has little influence on the spread of hepatitis C.

(2)From the result of the sensitivity analysis of ${\mathcal {R}_{0}}$, we can find that parameters *l,m*,*n* have negligible influence on the spread of hepatitis C, compared to the most sensitive parameters *β*_2_,*β*_1_,*σ*,*α* (see Table 3 for details).

(3)From the sensitivity analysis of the endemic equilibrium, we can see that parameters *l,m*,*n* are not sensitive to it. So reducing the transmission rate of vertical infection has no influence on controlling the scale of patients with HCV (see Table 4 for details).

Therefore, it is reasonable to ignore vertical infection in the existing hepatitis C dynamics models [10, 15, 18–24]. Contact transmission (such as injecting contaminated blood, using public syringe, sexual behavior and so on) is the main factor for the epidemic of the hepatitis C in China, the reasons are as follows:

(1)${\bar R_{01}} = 0.6759, {\bar R_{02}} = 0.8529, {\bar R_{03}} = 0.1303$ represent the average contribution of the exposed infection, the acute infection and the chronic infection to the basic reproduction number (${\mathcal {R}_{0}}$), respectively. We can find that contact transmission has great effect on the spread of hepatitis C.

(2)From the result of the sensitivity analysis of ${\mathcal {R}_{0}}$, we can find that the sensitive indexes of the parameters *β*_1_ (the second), *β*_2_ (the first), *β*_3_ (the fifth) are extremely large (see Table 3 for details).

(3)From the result of the sensitivity analysis of the endemic equilibrium, we can see that the parameters *β*_1_,*β*_2_,*β*_3_ are sensitive to it. So reducing the transmission rate *β*_1_,*β*_2_,*β*_3_ can effectively control the scale of patients with hepatitis C (see Table 4 for details).

In addition, the exposed and the acute infection tend to be asymptomatic, so the susceptible have more chance to contact them. Therefore, contact transmission is the main reason for the epidemic of hepatitis C in China.

## Conclusions

In this paper, we constructed an *SEI*_*a*_*I*_*c*_*TR* dynamic model for hepatitis C transmission based on the reported data from China’s CDC to search the most influential parameters. From the last line in Table 2, the basic reproductive number ${\mathcal {R}_{0}}$ in each year is larger than 1. Thus, we conclude that HCV will persist in China under the current conditions. As a matter of fact, there is no effective vaccine for HCV, but if we can provide some preventive measures to control the HCV, it will be very meaningful.

**Fig. 3:**
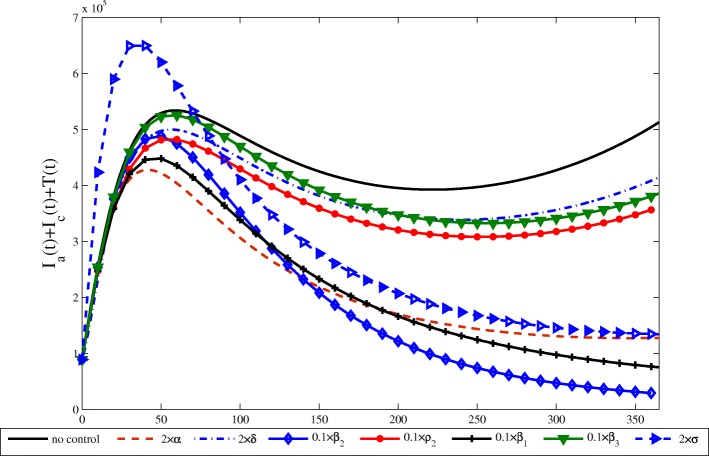
Simulation of the sum of not hospitalized infectious *I*_*a*_(*t*)+*I*_*c*_(*t*) and hospitalized infectious *T*(*t*) with all parameters, 2*α*=2×0.0162=0.0324,2*δ*=2×0.1=0.2,0.1*β*_2_=0.1×0.0246=0.00246,0.1*ρ*_2_=0.1×0.87=0.087,0.1*β*_1_=0.1×0.0223=0.00223,0.1*β*_3_=0.1×0.022=0.0022,2*σ*=2×0.0272=0.0544 from the seventh column of Table 2, respectively, when one parameter takes a specific value, the other parameters take the value of the seventh column in Table 2

Next, we selected the data of 2016 to simulate the future prevalence trend of hepatitis C in China under various circumstances, and the results were shown in Fig. 3. We can observe that *β*_2_,*β*_1_,*α* and *σ* are the most sensitive parameters comparing with the others because just slight changes can achieve the goal of control. These existing measures to control and prevent HCV can be essentially attributed to how to reduce *β*_2_ and *β*_1_. Based on the discussion in this paper, it is vitally important not only to reduce *β*_2_ and *β*_1_, but also to increase *α* and *σ*. In addition, it is more effectively to reduce *β*_2_ and *β*_1_ than to reduce *β*_3_ precisely because chronic patients will pay more attention to the contact with others and do a good job of protection than those who do not show symptoms in the incubation and acute period.

Based on the above analysis, we propose some preventive measures as follows:

(1) It can control the spread of the HCV by reducing infection rate of contacting with the exposed and the acutely infected to the susceptible (*β*_1_ and *β*_2_) (see Fig. 3). Therefore, it is vital to advocate public education so that we can understand the spread of HCV well and reduce the probability of contacting with the patients. For example, avoid unnecessary injection, transfusion and using of blood products unless go to formally medical health institutions. It is necessary to disinfect strictly for bloody items and the humoral pollutants. Stay away from drugs and educate intravenous drug users to let them know the harm of impurity injection and give them some advice about drug rehabilitation.

(2) It can control the spread of HCV at a lower level by shortening the diagnosis time of acute infection (1/*α*) and the hesitant time for being treated of chronically infected patients (1/*δ*) (see Fig. 3). That is, improve the transition rates of the acute (*α*) and chronic infection patients (*δ*), especially for *α*, which has extremely high sensitivity not only to the basic reproduction number but also to the endemic equilibrium. If we often do exercise to improve our immunity, even if we are infected by HCV, we can restore health by autoimmunity. Check your body regularly, and hospital treatment can prevent the disease from aggravation. Although some HCV patients will recover after a period of oral medication at home, it is still necessary to encourage more chronic patients to receive treatment in hospital as quickly as possible, after all, it is more likely to recover and it could contact with less patients in the process of rehabilitation, so that the risk of being infected is also smaller for the susceptible.

(3) It can effectively control the spread of HCV by reducing the diagnosis time of exposed (1/*σ*), i.e., improve the rate of progressing to acute stage from the exposed stage (*σ*) (see Fig. 3). Thence, once we fell uncomfortable, we should go to a hospital for diagnosis in time, because the earlier you detect of the illness and treat, the more possibility you can recover [36].

(4) Reduce the proportion of chronic infection from acutely infection population *ρ*_2_ (see Fig. 3). From Tables 3 and 4, we can see that it is very sensitive to the basic reproduction number and the endemic equilibrium. So it is meaningful to received timely treatment, which can reduce the source of infection. Because 70% to 80% patients are asymptomatic [13, 14], it is difficult to diagnose acute HCV infection. But some studies suggest that acute infection stage is very sensitive to treatment, and it is an unique opportunity to prevent the evolution of chronic infection [15].

(5) It can control the number of patients in a relatively small size by improving recovery rate of hospitalization *η*_1_. It is not sensitive to the basic reproduction number, but it is sensitive to the endemic equilibrium. It need not only patients cooperate with treatment actively but also relevant departments study new and effective medicine for the treatment of HCV [37–39]. It can improve the recovery rate of patients.

In a word, if we can implement these control measures, HCV will be controlled well, and with the time flies, the number of patients will decrease.

## Appendix

By the parameters value of 2015 given in Table 2, we can calculate the endemic equilibrium values:

*P*^∗^ = (*S*^∗^,*E*^∗^,*I*_*a*_^∗^,*I*_*c*_^∗^,*T*^∗^,*R*^∗^)=(184287929.9,575029.5,783783.9,83492.0,1880.2,140659573.5).

The variables (*S,E*,*I*_*a*_,*I*_*c*_,*T,R*) have been replaced by *x*_1_,*x*_2_,*x*_3_,*x*_4_,*x*_5_,*x*_6_; the parameters *l,m*,*n*,*β*_1_,*β*_2_,*β*_3_,*σ*,*α*,*ρ*_1_,*ρ*_2_,*δ*,*η*_1_,*λ*,*p*_1_,*γ*,*Λ*,*μ*,*d*_1_,*d*_2_ by *y*_1_, *y*_2_, *y*_3_, *y*_4_,*y*_5_,*y*_6_,*y*_7_,*y*_8_,*y*_9_,*y*_10_,*y*_11_,*y*_12_,*y*_13_,*y*_14_,*y*_15_,*y*_16_,*y*_17_,*y*_18_,*y*_19_; the point of endemic equilibrium $({S^{*}}, {E^{*}}, I_{a}^{*}, I_{c}^{*}, {T^{*}}, {R^{*}})$ by $x_{1}^{*},x_{2}^{*},x_{3}^{*},x_{4}^{*},x_{5}^{*},x_{6}^{*}$ and six equilibrium equations of the model by

$${f_{i}}({x_{1}},...,{x_{6}};{y_{1}},...,{y_{19}}) = 0,i = 1,2,3,4,5,6. $$

$${{} \begin{aligned} {f_{1}}({x_{1}},...,{x_{6}};{y_{1}},...,{y_{19}}) &= {y_{16}} - {y_{17}}{y_{1}}{x_{2}} - {y_{17}}{y_{2}}{x_{3}} - {y_{17}}{y_{3}}{x_{4}} \\&- \frac{{({y_{4}}{x_{2}} + {y_{5}}{x_{3}} + {y_{6}}{x_{4}}){x_{1}}}}{{{x_{1}} + {x_{2}} + {x_{3}} + {x_{4}} + {x_{5}} + {x_{6}}}} \\&\quad+ {y_{15}}{x_{6}} - {y_{17}}{x_{1}} = 0, \end{aligned}} $$

$${\begin{aligned} {f_{2}}({x_{1}},...,{x_{6}};{y_{1}},...,{y_{19}}) &\,=\, \frac{{({y_{4}}{x_{2}} + {y_{5}}{x_{3}} + {y_{6}}{x_{4}}){x_{1}}}}{{{x_{1}} + {x_{2}} + {x_{3}} + {x_{4}} + {x_{5}} + {x_{6}}}} \,-\, {y_{7}}{x_{2}} - {y_{17}}{x_{2}} \\&+ {y_{17}}{y_{1}}{x_{2}} + {y_{17}}{y_{2}}{x_{3}} + {y_{17}}{y_{3}}{x_{4}} = 0, \end{aligned}} $$

$${{} \begin{aligned} {f_{3}}({x_{1}},...,{x_{6}};{y_{1}},...,{y_{19}}) = {y_{7}}{x_{2}} - {y_{8}}{x_{3}} - {y_{17}}{x_{3}} - {y_{18}}{x_{3}} = 0, \end{aligned}} $$

$${{}\begin{aligned} {f_{4}}({x_{1}},...,{x_{6}};{y_{1}},...,{y_{19}}) &= {y_{10}}{y_{8}}{x_{3}} - {y_{11}}{x_{4}} \\ &+ (1 \,-\, {y_{12}}){y_{13}}{x_{5}}\! - \!{y_{17}}{x_{4}}\! - \!{y_{19}}{x_{4}} = 0, \end{aligned}} $$

$${{} \begin{aligned} {f_{5}}({x_{1}},...,{x_{6}};{y_{1}},...,{y_{19}}) &= (1 - {y_{9}} - {y_{10}}){y_{8}}{x_{3}} \\&+ (1 - {y_{9}}){y_{11}}{x_{4}} \,-\, {y_{13}}{x_{5}} \,-\, {y_{17}}{x_{5}} = 0, \end{aligned}} $$

and

$${\begin{aligned} {f_{6}}({x_{1}},...,{x_{6}};{y_{1}},...,{y_{19}}) &= {y_{14}}{y_{11}}{x_{4}} + {y_{9}}{y_{8}}{x_{3}} + {y_{12}}{y_{13}}{x_{5}} \\&- ({y_{15}} + {y_{17}}){x_{6}} = 0. ~~~~~~~~~~~~~~~~~~~~~~~~~~~~~ \end{aligned}} $$

Let *AX*_*j*_=*K*_*j*_ be the system of equations where

$${\begin{aligned} A& = \left[ {\begin{array}{*{20}{c}} {{a_{11}}}&{{a_{12}}}&{{a_{13}}}&{{a_{14}}}&{{a_{15}}}&{{a_{16}}}\\ {{a_{21}}}&{{a_{22}}}&{{a_{23}}}&{{a_{24}}}&{{a_{25}}}&{{a_{26}}}\\ {{a_{31}}}&{{a_{32}}}&{{a_{33}}}&{{a_{34}}}&{{a_{35}}}&{{a_{36}}}\\ {{a_{41}}}&{{a_{42}}}&{{a_{43}}}&{{a_{44}}}&{{a_{45}}}&{{a_{46}}}\\ {{a_{51}}}&{{a_{52}}}&{{a_{53}}}&{{a_{54}}}&{{a_{55}}}&{{a_{56}}}\\ {{a_{61}}}&{{a_{62}}}&{{a_{63}}}&{{a_{64}}}&{{a_{65}}}&{{a_{66}}} \end{array}} \right];\\&\quad {X_{j}} = \left[ {\begin{array}{*{20}{c}} {\frac{{\partial x_{1}^{*}}}{{\partial {y_{j}}}}}\\ {\frac{{\partial x_{2}^{*}}}{{\partial {y_{j}}}}}\\ {\frac{{\partial x_{3}^{*}}}{{\partial {y_{j}}}}}\\ {\frac{{\partial x_{4}^{*}}}{{\partial {y_{j}}}}}\\ {\frac{{\partial x_{5}^{*}}}{{\partial {y_{j}}}}}\\ {\frac{{\partial x_{6}^{*}}}{{\partial {y_{j}}}}} \end{array}} \right];{K_{j}} = \left[ {\begin{array}{*{20}{c}} { - \frac{{\partial {f_{1}}}}{{\partial {y_{j}}}}}\\ { - \frac{{\partial {f_{2}}}}{{\partial {y_{j}}}}}\\ { - \frac{{\partial {f_{3}}}}{{\partial {y_{j}}}}}\\ { - \frac{{\partial {f_{4}}}}{{\partial {y_{j}}}}}\\ { - \frac{{\partial {f_{5}}}}{{\partial {y_{j}}}}}\\ { - \frac{{\partial {f_{6}}}}{{\partial {y_{j}}}}} \end{array}} \right]; \end{aligned}} $$

$${\begin{aligned} \begin{array}{l} {a_{11}} = \frac{{{x_{1}}\left({{x_{2}}{y_{4}} + {x_{3}}{y_{5}} + {x_{4}}{y_{6}}} \right)}}{{{{({x_{1}} + {x_{2}} + {x_{3}} + {x_{4}} + {x_{5}} + {x_{6}})}^{2}}}} - \frac{{{x_{2}}{y_{4}} + {x_{3}}{y_{5}} + {x_{4}}{y_{6}}}}{{{x_{1}} + {x_{2}} + {x_{3}} + {x_{4}} + {x_{5}} + {x_{6}}}} - {y_{17}},\\ {a_{12}} = \frac{{{x_{1}}\left({{x_{2}}{y_{4}} + {x_{3}}{y_{5}} + {x_{4}}{y_{6}}} \right)}}{{{{({x_{1}} + {x_{2}} + {x_{3}} + {x_{4}} + {x_{5}} + {x_{6}})}^{2}}}} - {y_{1}}{y_{17}} - \frac{{{x_{1}}{y_{4}}}}{{{x_{1}} + {x_{2}} + {x_{3}} + {x_{4}} + {x_{5}} + {x_{6}}}},\\ {a_{13}} = \frac{{{x_{1}}\left({{x_{2}}{y_{4}} + {x_{3}}{y_{5}} + {x_{4}}{y_{6}}} \right)}}{{{{({x_{1}} + {x_{2}} + {x_{3}} + {x_{4}} + {x_{5}} + {x_{6}})}^{2}}}} - {y_{2}}{y_{17}} - \frac{{{x_{1}}{y_{5}}}}{{{x_{1}} + {x_{2}} + {x_{3}} + {x_{4}} + {x_{5}} + {x_{6}}}},\\ {a_{14}} = \frac{{{x_{1}}\left({{x_{2}}{y_{4}} + {x_{3}}{y_{5}} + {x_{4}}{y_{6}}} \right)}}{{{{({x_{1}} + {x_{2}} + {x_{3}} + {x_{4}} + {x_{5}} + {x_{6}})}^{2}}}} - {y_{3}}{y_{17}} - \frac{{{x_{1}}{y_{6}}}}{{{x_{1}} + {x_{2}} + {x_{3}} + {x_{4}} + {x_{5}} + {x_{6}}}},\\ {a_{15}} = \frac{{{x_{1}}\left({{x_{2}}{y_{4}} + {x_{3}}{y_{5}} + {x_{4}}{y_{6}}} \right)}}{{{{({x_{1}} + {x_{2}} + {x_{3}} + {x_{4}} + {x_{5}} + {x_{6}})}^{2}}}}, {a_{16}} = {y_{15}} + \frac{{{x_{1}}\left({{x_{2}}{y_{4}} + {x_{3}}{y_{5}} + {x_{4}}{y_{6}}} \right)}}{{{{({x_{1}} + {x_{2}} + {x_{3}} + {x_{4}} + {x_{5}} + {x_{6}})}^{2}}}},\\ {a_{21}} = \frac{{{x_{2}}{y_{4}} + {x_{3}}{y_{5}} + {x_{4}}{y_{6}}}}{{{x_{1}} + {x_{2}} + {x_{3}} + {x_{4}} + {x_{5}} + {x_{6}}}} - \frac{{{x_{1}}\left({{x_{2}}{y_{4}} + {x_{3}}{y_{5}} + {x_{4}}{y_{6}}} \right)}}{{{{({x_{1}} + {x_{2}} + {x_{3}} + {x_{4}} + {x_{5}} + {x_{6}})}^{2}}}},\\ {a_{22}} \,=\, {y_{1}}{y_{17}} \!\,-\, {y_{17}} \,-\, {y_{7}} \,-\, \frac{{{x_{1}}\left({{x_{2}}{y_{4}} + {x_{3}}{y_{5}} + {x_{4}}{y_{6}}} \right)}}{{{{({x_{1}} + {x_{2}} + {x_{3}} + {x_{4}} + {x_{5}} + {x_{6}})}^{2}}}} \,+\, \frac{{{x_{1}}{y_{4}}}}{{{x_{1}} \,+\, {x_{2}} + {x_{3}} + {x_{4}} + {x_{5}} + {x_{6}}}},\\ {a_{23}} = {y_{2}}{y_{17}} - \frac{{{x_{1}}\left({{x_{2}}{y_{4}} + {x_{3}}{y_{5}} + {x_{4}}{y_{6}}} \right)}}{{{{({x_{1}} + {x_{2}} + {x_{3}} + {x_{4}} + {x_{5}} + {x_{6}})}^{2}}}} + \frac{{{x_{1}}{y_{5}}}}{{{x_{1}} + {x_{2}} + {x_{3}} + {x_{4}} + {x_{5}} + {x_{6}}}},\\ {a_{24}} = {y_{3}}{y_{17}} - \frac{{{x_{1}}\left({{x_{2}}{y_{4}} + {x_{3}}{y_{5}} + {x_{4}}{y_{6}}} \right)}}{{{{({x_{1}} + {x_{2}} + {x_{3}} + {x_{4}} + {x_{5}} + {x_{6}})}^{2}}}} + \frac{{{x_{1}}{y_{6}}}}{{{x_{1}} + {x_{2}} + {x_{3}} + {x_{4}} + {x_{5}} + {x_{6}}}},\\ {a_{25}} = - \frac{{{x_{1}}\left({{x_{2}}{y_{4}} + {x_{3}}{y_{5}} + {x_{4}}{y_{6}}} \right)}}{{{{({x_{1}} + {x_{2}} + {x_{3}} + {x_{4}} + {x_{5}} + {x_{6}})}^{2}}}}, {a_{26}} = - \frac{{{x_{1}}\left({{x_{2}}{y_{4}} + {x_{3}}{y_{5}} + {x_{4}}{y_{6}}} \right)}}{{{{({x_{1}} + {x_{2}} + {x_{3}} + {x_{4}} + {x_{5}} + {x_{6}})}^{2}}}},\\ {a_{31}} = 0, {a_{32}} = {y_{7}}, {a_{33}} = - {y_{8}} - {y_{17}} - {y_{18}},{a_{34}} = 0,{a_{35}} \\\quad\quad= 0,{a_{36}} = 0,{a_{41}} = 0,{a_{42}} = 0,\\ {a_{43}} = {y_{8}}{y_{10}},{a_{44}} = - {y_{11}} - {y_{17}} - {y_{19}},{a_{45}} = - {y_{13}}({y_{12}} - 1),{a_{46}} \\\quad\quad= 0,{a_{51}} = 0,{a_{52}} = 0,\\ {a_{53}} = - {y_{8}}({y_{9}} + {y_{10}} - 1),{a_{54}} = - {y_{11}}({y_{9}} - 1),{a_{55}} = - {y_{13}} - {y_{17}},{a_{56}} \\ \quad\quad= 0,{a_{61}} = 0,{a_{62}} = 0,\\ {a_{63}} = {y_{8}}{y_{9}},{a_{64}} = {y_{11}}{y_{14}},{a_{65}} = {y_{12}}{y_{13}},{a_{66}} = - {y_{15}} - {y_{17}}. \end{array} \end{aligned}} $$

Finally, the sensitivity index of the point of endemic equilibrium, $x_{i}^{*}$ to the parameter, *y*_*j*_ is given by $\frac {{\partial x_{i}^{*}}}{{\partial {y_{j}}}}\frac {{{y_{j}}}}{{x_{i}^{*}}}$ for 1≤*i*≤6 and 1≤*j*≤16.

## Data Availability

The data that support the findings of this study are available from the China Center for Disease Control and Prevention (China’s CDC) (http://www.nhc.gov.cn/jkj/s2907/new_list.shtml?tdsourcetag=s_pcqq_aiomsg), these network direct data are completely open, and we count these data month by month.

## Abbreviations

China’s CDC: Chinese center for disease control and prevention
HCC: Hepatocellular carcinoma
HCV: Hepatitis C virus
IDUs: Injecting drug users
RNA: Ribonucleic acid
*SEI*_*a*_*I*_*c*_*TR*: Susceptible-exposed-acute infection-chronic infection-treated-recovered
SQP: Sequence quadratic program
WHO: The world health organization

## References

[1] Hepatitis C. http://www.who.int/news-room/fact-sheets/detail/hepatitis-c. Accessed 9 July 2019.

[2] Edlin BR, Eckhardt BJ, Shu MA, Holmberg SD, Swan T (2015). Toward a more accurate estimate of the prevalence of hepatitis c in the united states. Hepatology.

[3] Surveillance for Viral Hepatitis-United States. 2016. https://www.cdc.gov/hepatitis/statistics/2016surveillance/pdfs/2016HepSurveillanceRpt.pdf. Accessed 11 Jan 2017.

[4] Hope VD, Eramova I, Capurro D, Donoghoe MC (2014). Prevalence and estimation of hepatitis B and C infections in the WHO European Region: a review of data focusing on the countries outside the European Union and the European Free Trade Association. Epidemiol Infect.

[5] Young J, Weis N, Hofer H, Irving W, Weiland O, Giostra E, Pascasio JM, Castells L, Prieto M, Postema R (2017). The effectiveness of daclatasvir based therapy in european patients with chronic hepatitis c and advanced liver disease. BMC Infect Dis.

[6] Razali K, Thein HH, Bell J, Cooper-Stanbury M, Dolan K, Dore G, George J, Kaldor J, Karvelas M, Li J (2007). Modelling the hepatitis c virus epidemic in australia. Drug Alcohol Depend.

[7] Dazley JS, Sriramulu LD, Slim J (2017). Decompensated hcv patients with co morbidities including hiv who are medically treated are shown to minimize decompensation related admissions and healthcare cost: A case series. J Infect Pub Health.

[8] Choo Q-L, Kuo G, Weiner AJ, Overby LR, Bradley DW, Houghton M (1989). Isolation of a cdna clone derived from a blood-borne non-a, non-b viral hepatitis genome. Science.

[9] Kuo G, Choo Q-L, Alter H, Gitnick G, Redeker A, Purcell R, Miyamura Te, Dienstag J, Alter M, Stevens C (1989). An assay for circulating antibodies to a major etiologic virus of human non-a, non-b hepatitis. Science.

[10] Martcheva M, Castillo-Chavez C (2003). Diseases with chronic stage in a population with varying size. Math Biosci.

[11] Shepard CW, Finelli L, Alter MJ (2005). Global epidemiology of hepatitis c virus infection. Lancet Infect Dis.

[12] What Is the Incubation Period for Hepatitis C? https://www.healthline.com/health/hepatitis-c-incubation-period. Accessed 27 Aug 2017.

[13] Chen SL, Morgan TR (2006). The natural history of hepatitis c virus (hcv) infection. Int J Med Sci.

[14] Alberti A, Chemello L, Benvegnù L (1999). Natural history of hepatitis c. J Hepatol.

[15] Das P, Mukherjee D, Sarkar A (2005). Analysis of a disease transmission model of hepatitis c. J Biol Syst.

[16] Tong MJ, El-Farra NS, Reikes AR, Co RL (1995). Clinical outcomes after transfusion-associated hepatitis c. N Engl J Med.

[17] Seeff LB (1997). Natural history of hepatitis c. Hepatology.

[18] Yuan J, Yang Z (2008). Global dynamics of an sei model with acute and chronic stages. J Comput Appl Math.

[19] Imran M, Hassan M, Dur-E-Ahmad M, Khan A (2013). A comparison of a deterministic and stochastic model for hepatitis c with an isolation stage. J Biol Dyn.

[20] Zeiler I, Langlands T, Murray JM, Ritter A (2010). Optimal targeting of hepatitis c virus treatment among injecting drug users to those not enrolled in methadone maintenance programs. Drug Alcohol Depend.

[21] Martin NK, Vickerman P, Hickman M (2011). Mathematical modelling of hepatitis c treatment for injecting drug users. J Theor Biol.

[22] Martin NK, Vickerman P, Foster GR, Hutchinson SJ, Goldberg DJ, Hickman M (2011). Can antiviral therapy for hepatitis c reduce the prevalence of hcv among injecting drug user populations? a modeling analysis of its prevention utility. J Hepatol.

[23] Zhang S, Zhou Y (2012). Dynamics and application of an epidemiological model for hepatitis c. Math Comput Model.

[24] Shen M, Xiao Y, Zhou W, Li Z (2015). Global dynamics and applications of an epidemiological model for hepatitis c virus transmission in china. Discret Dyn Nat Soc.

[25] Prevention and Control of Infectious Diseases (in Chinese). http://www.nhc.gov.cn/jkj/s2907/new_list.shtml?tdsourcetag=s_pcqq_aiomsg. Accessed 4 Aug 2019.

[26] Van den Driessche P, Watmough J (2002). Reproduction numbers and sub-threshold endemic equilibria for compartmental models of disease transmission. Math Biosci.

[27] National Bureau of Statistics of China (in Chinese). http://data.stats.gov.cn/easyquery.htm?cn=C01. Accessed 4 Aug 2019.

[28] Zhang X, Zhao Y, Neumann AU (2010). Partial immunity and vaccination for influenza. J Comput Biol.

[29] Li Y, Wang L, Pang L, Liu S (2016). The data fitting and optimal control of a hand, foot and mouth disease (hfmd) model with stage structure. Appl Math Comput.

[30] Information on Human Infection with H7N9 Avian Influenza (5 April 2013) (in Chinese). http://www.gov.cn/gzdt/2013-04/06/content_2370976.htm. Accessed 6 Apr 2013.

[31] Xinhua Net, “A 7.0-magnitude Strong Earthquake Hit Ya’an, in Sichuan Province” (in Chinese). http://www.xinhuanet.com/politics/earthquake/index.htm. Accessed 20 Apr 2013.

[32] Samsuzzoha M, Singh M, Lucy D (2013). Uncertainty and sensitivity analysis of the basic reproduction number of a vaccinated epidemic model of influenza. Appl Math Model.

[33] Chitnis N, Hyman JM, Cushing JM (2008). Determining important parameters in the spread of malaria through the sensitivity analysis of a mathematical model. Bull Math Biol.

[34] Cox AL (2015). Global control of hepatitis c virus. Science.

[35] Seeff LB (1999). Natural history of hepatitis c. Am J Med.

[36] Cohen J (2012). Calling all baby boomers: get your hepatitis C test. Am Assoc Adv Sci.

[37] Enserink M (2011). First specific drugs raise hopes for hepatitis C. Am Assoc Adv Sci.

[38] Hill A, Cooke G (2014). Hepatitis c can be cured globally, but at what cost?. Science.

[39] Cohen J (2012). Despite setbacks, optimism on drugs for hepatitis C. Am Assoc Adv Sci.

